# Piezoelectric Transformer-Based High-Voltage Pulse Generator Using Wide-Bandgap Semiconductors for Medical Electroporation Therapy

**DOI:** 10.1007/s10439-023-03319-6

**Published:** 2023-07-19

**Authors:** Ajay M. Chole, Maeve Duffy

**Affiliations:** Power Electronics Research Centre, University of Galway, Galway, H91 HX31 Ireland

**Keywords:** Piezoelectric transformer, Wide-bandgap power semiconductors, High-voltage pulse generator, Electroporation

## Abstract

In this paper, a new application of Piezoelectric Transformer (PT)-based power converters to generate high-voltage (HV) bipolar pulses for medical electroporation therapy is proposed. In particular, PT-based power conversion is investigated as an alternative to magnetics-based approaches of generating HV from a relatively low-voltage (LV) input source for application in electroporation therapy. The detailed PT-based system design and selection of wide bandgap semiconductor switches such as GaN FETs, high-voltage SiC diodes and SiC MOSFETs, as well as simulation results to demonstrate proof-of-concept using LTSpice are presented. Preliminary experimental results of the PT-based capacitor charger are shown, and work is ongoing to develop a complete hardware prototype of the proposed HV pulse generator.

## Introduction

The permeability of cell membranes and tissues is enhanced when exposed to pulsed electric fields of a high amplitude and short duration. This enhancement in permeability is attributed to the momentary formation of aqueous pores in the cell membrane, a process known as electroporation. Electroporation is a well-established technology in the field of medicine and biotechnology for applications such as the introduction of molecules into cells, cell-fusion, electrochemotherapy, tissue ablation, gene-therapy, and sterilization of water and liquid food [5].

**Fig. 1:**
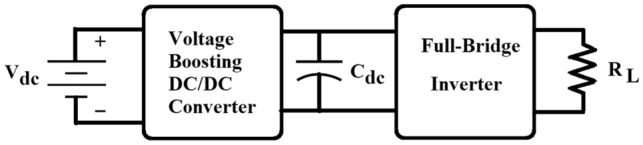
Magnetics based HV pulse generator using capacitor charging principle

Electroporation systems typically consist of at least two stages of power conversion as shown in Fig. 1. The first stage generally consists of a high-step-up DC/DC converter that charges a DC-link capacitor to a high voltage (HV, typically several kV) from a low-voltage (LV) source. The second stage is an inverter required to convert the DC voltage to a train of high-frequency HV pulses. The main focus of this paper is on the first stage; i.e. high step-up DC/DC converter.

Voltage boosting DC/DC converters for capacitor charging application proposed in the literature are Boost, Buck-Boost, Flyback (isolated Buck-Boost) and LLC resonant converters [1], all of which require an inductor and/or transformer as an energy storage element. Similarly, most step-up DC/DC power conversion stages proposed for HV pulse generators for medical electroporation make use of either an inductor and/or a transformer [1, 5, 37].

Pulse generators based on topologies such as the Solid-State Marx Generator (SSMG) [7, 8, 26, 27, 32, 35], Capacitor-Diode Voltage Multiplier (CDVM) [28, 30], Switched Capacitor Voltage Multiplier (SCVM) [12, 14, 22] have no magnetic components, but may become bulky for high step-up ratios as a large number of capacitor stages and therefore switches with different voltage ratings would be needed. This work proposes a new alternative to magnetics-based HV pulse generators for medical electroporation, which may also compete with magnetic-less HV pulse generators such as SSMG, CDVM or SCVM.

PTs are a special type of transformers which are based on vibration rather than electromagnetics as a coupling medium to transfer input electrical energy to mechanical energy and then again back to electrical energy at the output with a different voltage amplitude [9, 11]. Compared to traditional electromagnetic transformers, PTs have advantages such as high power-density, low-electromagnetic interference (EMI), reduced weight, high-galvanic isolation, incombustibility, and comparable efficiency, particularly for high voltage step-up applications. For instance, a PT-based high-voltage CCFL backlight inverter was very popular for LCD displays in 2000-2010 [9]_._ The use of PTs in high-voltage DC capacitor charging has been previously demonstrated with a relatively small capacitance (less than 1 µF) for applications such as Electro-Active Polymer (EAP) actuators [2]_,_ automobile air-bag ignition systems [41] and pulsed-power systems [23].

In the proposed application of medical electroporation, the size of the DC capacitor is substantially larger, typically 100’s μF depending on the required pulse-width, pulse-number, acceptable voltage drop, pulse-power, losses and repetition frequency during a typical therapy session. Therefore, the PT-based converter power delivery, efficiency and dynamic response all need to be carefully considered to provide an optimized solution. The objective of this work is to explore and extend the application of PTs to generate HV pulses for electroporation at a better performance, size, weight, and cost compared to existing electromagnetic converter solutions [25, 29, 39].

A complete PT-based HV pulse generator with 24 *V*_dc_ input that outputs bipolar pulses up to 2 kV typical of those used in medical electroporation is designed and investigated using LTSpice simulation. A small-scale hardware prototype of a PT-based capacitor charger circuit has been built and tested using general-purpose low-cost standard silicon components to validate the concept. The experimental results successfully demonstrate the charging operation and verify the potential of the PT-based converter in medical electroporation therapy.

Work is ongoing to construct a full hardware prototype (i.e. PT-based capacitor charger and HV inverter circuits both working together) to determine the performance, size, and weight of the proposed solution, for comparison with commercially available medical electroporation power supplies

## PT-Based HV Pulse Generator for Electroporation

**Fig. 2:**
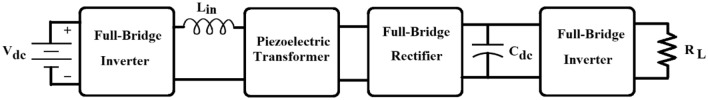
PT-based HV pulse generator architecture for Electroporation

Figure 2 shows a block diagram of the proposed HV pulse generator based on a step-up PT. It consists of a low-input voltage DC source followed by a DC-AC inverter stage to drive the PT close to its resonant frequency. The amplified high-frequency PT output voltage is rectified and supplied to a DC-link capacitor, *C*_dc_, which charges to the required high-voltage (HV). The charging time depends on the capacitance value and the rated power of the PT. Once the DC-link capacitor is charged, the input inverter is switched OFF and the DC-link voltage is applied in pulses to the electrodes (represented here as an effective load resistance, R_L_), through an output full-bridge inverter that switches at the required pulse-width and frequency.

### Full-Bridge LV Inverter

To obtain optimal performance, the PT should be driven with an AC sinusoidal voltage to extract full rated power at the output. Low-voltage (LV) inverter topologies proposed in the literature include a half-bridge [10], push–pull [36], single-switch class-E [20] or a full-bridge inverter [33], all of which generate square-wave voltages. In this paper, maximum power is transferred in both halves of the input AC cycle to take full advantage of commercially available PTs to fast-charge the HV capacitor, using a full-bridge inverter with Gallium Nitride (GaN) FETs. It is anticipated that the use of GaN devices will result in higher conversion efficiency and smaller converter size compared to standard silicon MOSFETs.

### Series Inductor

If a square-wave voltage is directly applied to the PT, spikes occur in each cycle of the source current due to the PT input capacitance. These spikes may be reduced by adding a series inductor to form a low-pass filter with the PT input capacitance. Inductor-less driving of PTs by achieving Zero Voltage Switching (ZVS) has been proposed in the literature. This would provide significant benefits in medical equipment applications, where the elimination of magnetic components is required to minimise EMI and switching losses. However, ZVS capable PTs require special consideration during their design [31] and currently they are not available commercially. As a commercial Rosen PT has been used to prove the concept in this paper, a low-pass filter series inductor, *L*_in_, at the PT input is required.

### Rosen Piezoelectric Transformer

**Fig. 3:**
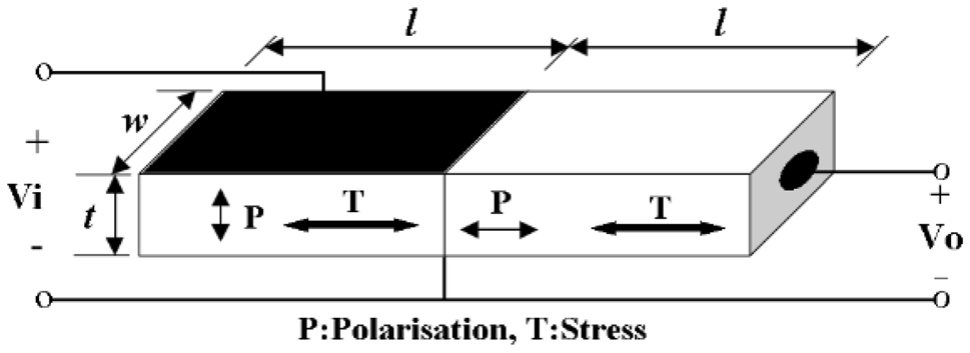
Basic structure of Rosen PT [11]

Invented by Dr. Rosen in the 1950s, the Rosen-PT structure shown in Fig. 3 is a combination of a transverse mode piezoelectric actuator at the input (driver) section and a longitudinal mode piezoelectric transducer at the output (generator) section, each of which dominates around the PT resonant frequency [11]. Because of the inherent high voltage gain associated with Rosen-PTs, they are referred as high-voltage PTs.

**Fig. 4:**
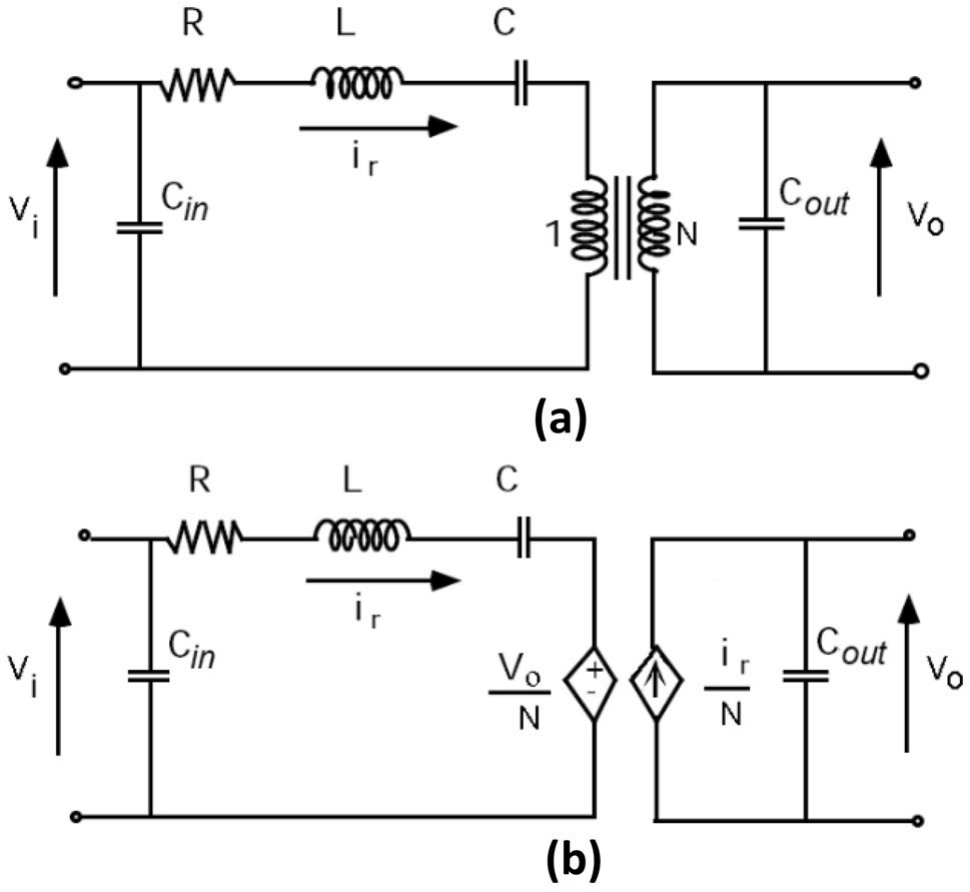
PT electrical equivalent circuit

The electrical equivalent circuit of the PT is shown in Fig. 4a, where *R* is the electrical equivalent of mechanical loss, *L* is an electrical equivalent of the PT mass, *C* is the equivalent of mechanical compliance, *C*_in_ and *C*_out_ represent static input and output capacitances, respectively. The series combination of *R, L* and *C* result in the circuit acting like a current source, which is more or less independent of the output load. The voltage transformation ratio is represented by an ideal electromagnetic transformer with turns ratio *N*. In Fig. 4b, the ideal transformer is replaced by interdependent voltage and current sources, which aids to speed-up computer simulations. It should be noted that this circuit only models the PT behaviour around primary resonance, and it does not account for higher order harmonic vibration modes.

### Full-Bridge HV Rectifier

Due to the resonant characteristics of the PT, its output response to the square-wave inverter is a sinusoidal AC voltage and requires a rectifier-stage to convert it to DC for HV energy-storage capacitor charging. In this work, a silicon carbide (SiC) diode-based full-bridge [40] rectifier stage is proposed to obtain higher converter efficiency and power-density when compared to standard silicon rectifiers.

### DC Link Capacitor

The DC-link capacitor is the main energy-storage component in the proposed system. The output of the HV rectifier is coupled to the DC-link capacitor *C*_dc_, which forms a source of energy to the next power conversion stage, i.e. full-bridge HV inverter. The value of capacitance must be chosen such that it will present a constant (stiff) DC source for repetitive short pulses with acceptable voltage drop as per the requirement of the aimed medical electroporation therapy session.

### Full-Bridge HV Inverter

The fully charged HV DC-link energy-storage capacitor is partially discharged to the electrodes through a HV inverter to produce high-amplitude bipolar-pulses at a required pulse-width, pulse-number, and repetition frequency. Recently, the requirement of very short HV pulses in the nanoseconds to microsecond range is growing for medical electroporation [5], therefore, a very fast HV semiconductor switch is required. Conventional silicon MOSFETs or IGBTs are slower and inefficient compared to latest generation SiC MOSFETs, hence in this research work, SiC MOSFETs are proposed for the output inverter.

## HV Pulse Generator Design and Simulation

**Fig. 5:**
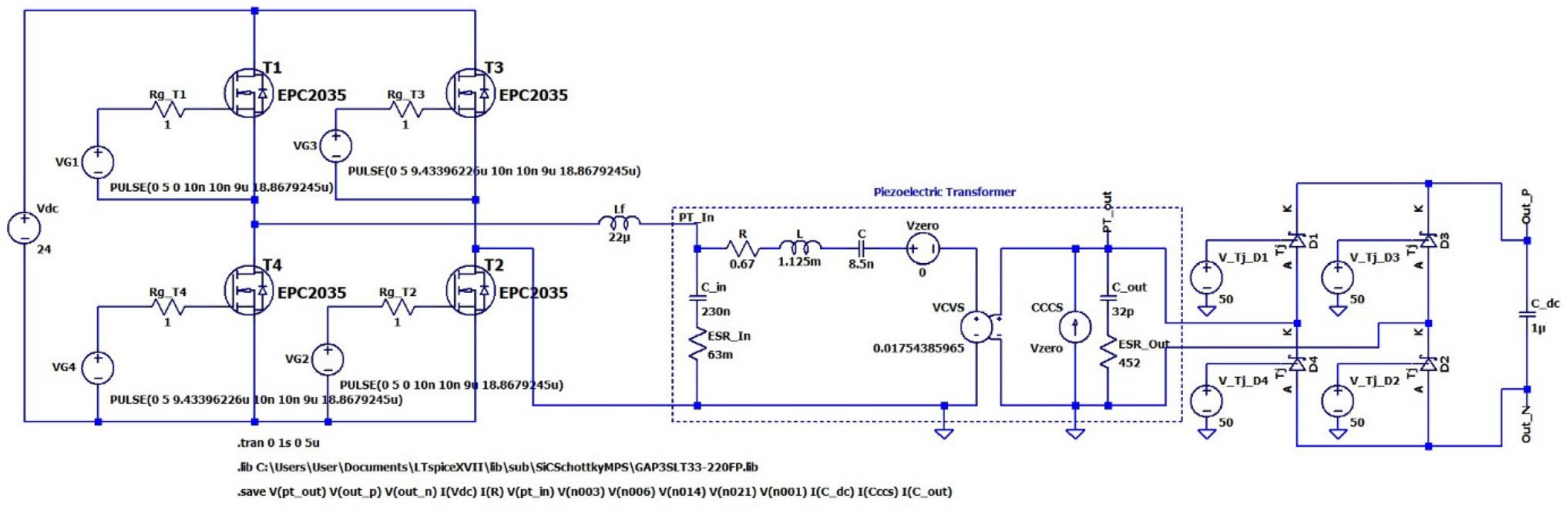
LTSpice schematic of the PT-based capacitor charger.

To demonstrate the proposed proof-of-concept, simulation results of a PT-based HV pulse generator for electroporation are presented in two stages, i.e., the HV capacitor charging circuit shown in Fig. 5 and a pulse-generator full-bridge SiC inverter shown later in Fig. 10.

The generator is designed to provide a typical electroporation treatment protocol consisting of eight bursts of 50 bipolar square wave pulses (1 µs positive polarity, 1 µs pause, 1 µs negative polarity and 1 µs pause) at a burst repetition rate of 1 Hz [24] from a 24 V DC supply. To maintain the applied voltage amplitude high enough over the treatment time, the DC link capacitor,* C*_dc_, needs to be chosen to limit its voltage drop according to:

1 $$\frac{1}{2}{C}_{\text{dc}}\left({{V}_{\text{u}}}^{2}-{{V}_{\text{l}}}^{2}\right)\ge \frac{{{V}_{l}}^{2}}{R}{N}_{\text{p}}{N}_{\text{b}}{t}_{\text{on}},$$

where *V*_u_ and *V*_l_ are the upper and lower voltage amplitudes, *R* is the effective resistance presented by the tissue at the electrodes, *N*_p_ is the number of pulses, *N*_b_ the number of bursts and *t*_on_ is the pulse duration. In this case, assuming a voltage drop of less than 3.5%, with a maximum voltage of 2 kV and a load-resistance of 50 Ω to emulate the in vivo resistance of tissue between needle electrodes (typically in the range of 40 Ω to 110 Ω [6]), *C*_dc_ is calculated as 433.3 μF. Accounting for practical capacitor tolerances, *C*_dc_ was implemented by connecting three 3.8 kV 165 µF film capacitors each with 1.6 mΩ equivalent series resistance, ESR (FFLR6Z1656KJE, Kyocera) in parallel to provide a nominal capacitance of 495 μF.

### Stage I: High Step-Up DC/DC Capacitor Charging Circuit

Figure 5 shows a circuit schematic of the PT-based capacitor charger implemented in LTSpice simulation using the commercial 6 W, 30 × 7.4 × 2.7 mm^3^, multilayer PT sample ELM-610 [13] considered in this work. This device was experimentally characterized and tested [4] to obtain equivalent circuit parameters of: *C*_in_ = 230 nF, *R* = 0.67 Ω, *L* = 1.125 mH, *C* = 8.5 nF, *N* = 57, *C*_out_ = 32 pF. The ESR values of input and output capacitances were estimated to be *R*_ESR_Cin_ = 63 mΩ and *R*_ESR_Cout_ = 452 Ω, respectively.

It should be noted that the range of commercial PTs available is quite limited, and therefore, circuit design is highly dependent on the particular models available. However, with increasing interest in magnetic-less power conversion solutions, it is expected that a wider range of models will become available in the near future.

**Fig. 6:**
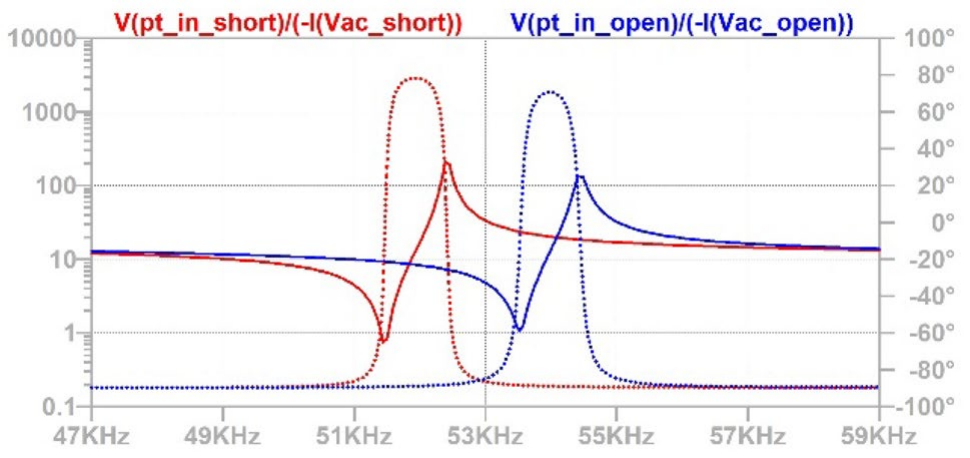
Input impedance of ELM-610

Using simulation of the given PT equivalent circuit parameters, the series resonant frequencies of 51.5 kHz and 53.5 kHz were determined from the PT input impedance response when short circuited and open circuited, respectively, as shown in Fig. 6. This correlates with the quoted resonant frequency of 55 kHz (± 3%) under open circuit, room temperature and small signal conditions [13]. In capacitor charging applications, short-circuit (SC) corresponds to the uncharged capacitor, while open-circuit (OC) represents a fully-charged capacitor; therefore, the operating frequency of the input GaN inverter, *f*, is chosen as an intermediate value of 53 kHz for initial investigation.

The GaN FET EPC2035 (*V*_DS_ = 60 V, *I*_DS_ = 1.7 A) is chosen to obtain a high-efficiency bridge-inverter (T_1_ to T_4_) at the low-voltage input side. The series inductor is chosen as 22 µH [38] to form a low-pass filter with the PT input capacitance with a cut-off frequency around 78 kHz. The SiC diode GAP3SLT33-220FP (*V*_RRM_ = 3.3 kV, *I*_F_ = 0.3 A) is chosen for the HV diode-bridge rectifier (D_1_ to D_4_). Note that the junction capacitances and leakage-resistances of the HV diode bridge, and the ESR/leakage resistance of HV capacitor affect the overall circuit gain, all of which are non-linear and therefore need to be accounted for in design.

**Fig. 7:**
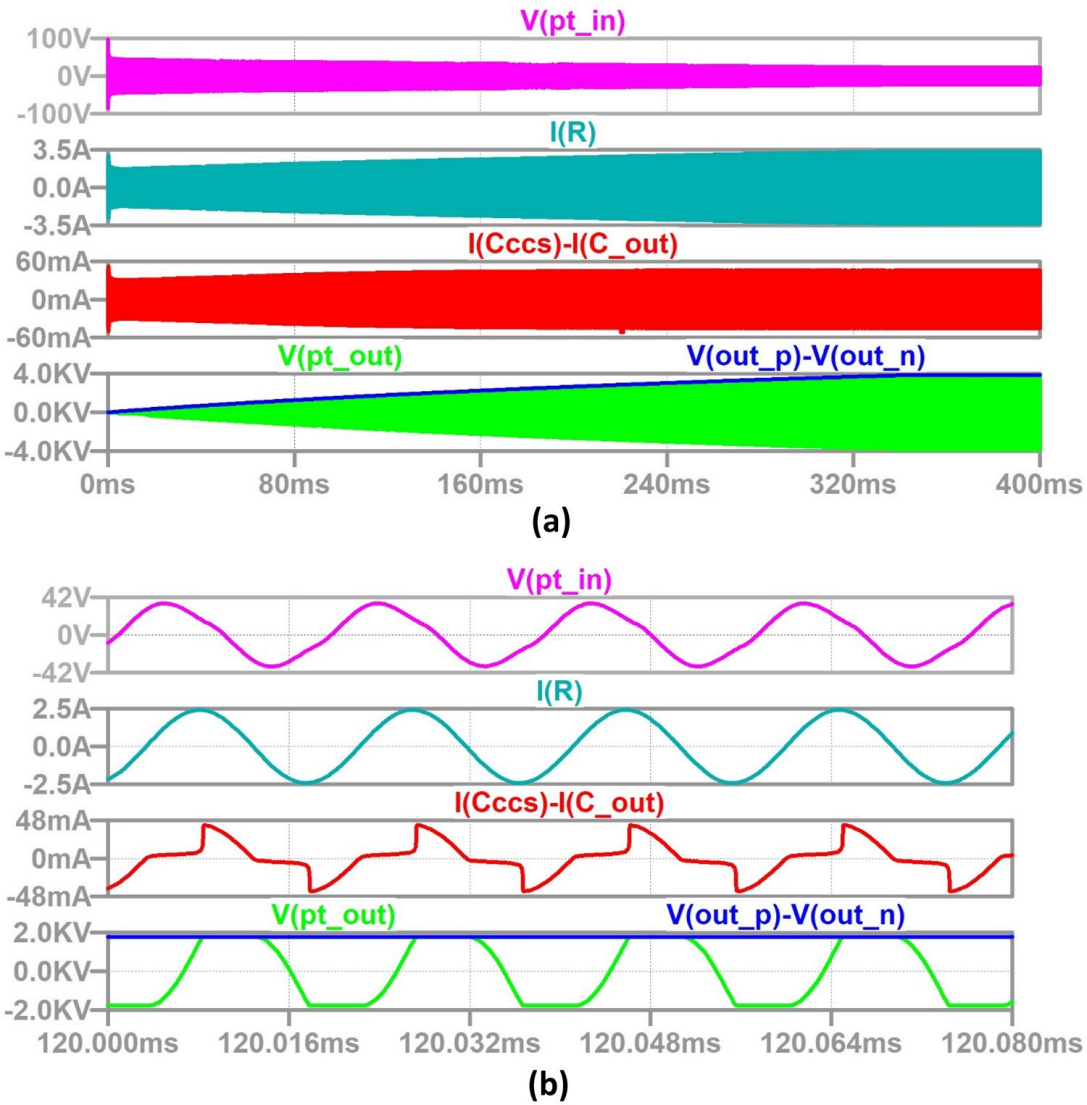
Waveforms of a PT-based capacitor charger for *C*_dc_ = 5µF **a** full charging cycle, **b** zoomed in at 120 ms (*V*_out_ =  ~ 2 kV): PT input voltage (magenta), resonant branch current (teal), PT output current (red), PT output voltage (green) and load capacitor voltage (blue).

**Fig. 8:**
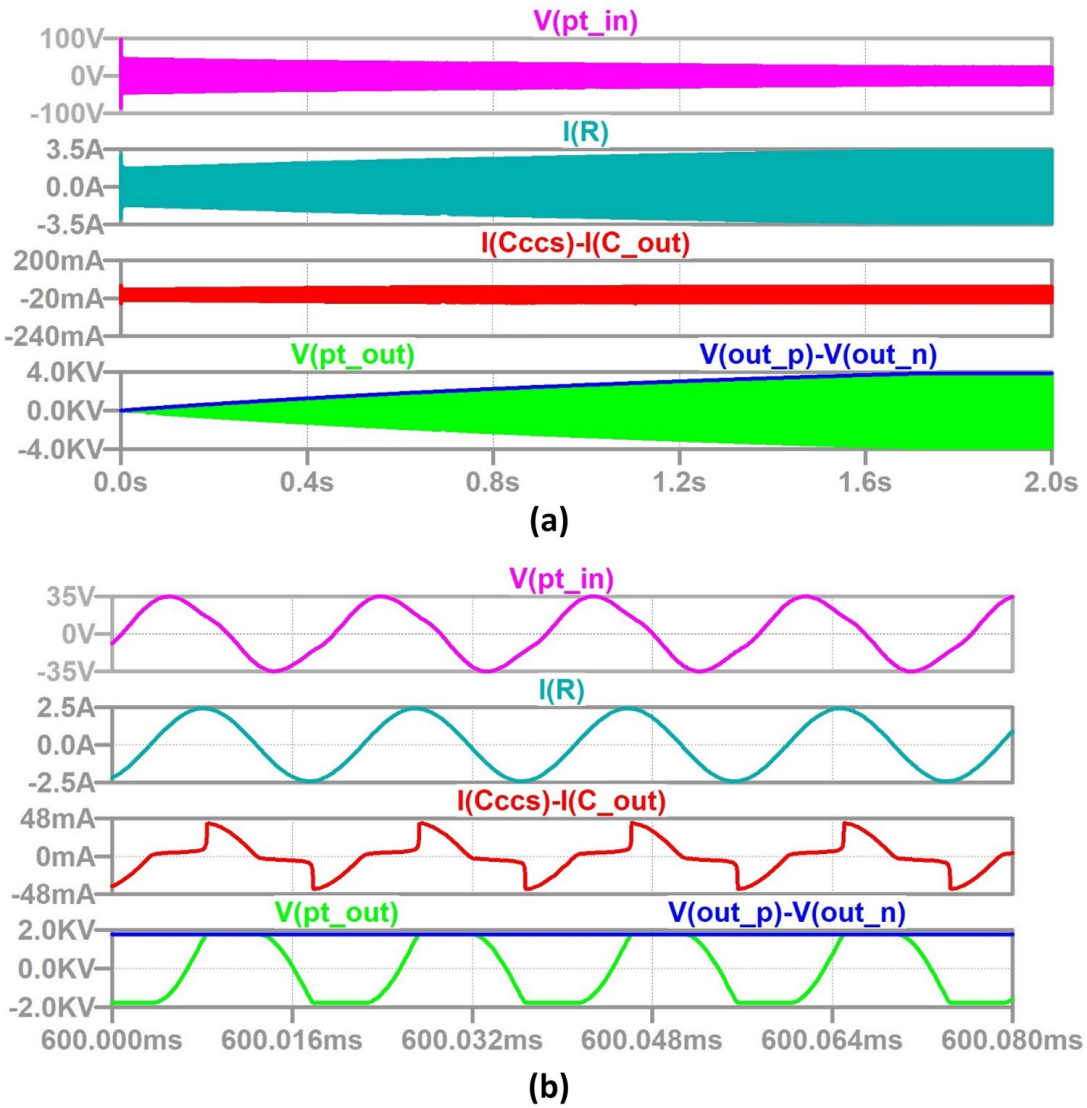
Waveforms of a PT-based capacitor charger for *C*_dc_ = 5 µF **a** full charging cycle, **b** zoomed in at 600 ms (*V*_out_ =  ~ 2 kV): PT input voltage (magenta), resonant branch current (teal), PT output current (red), PT output voltage (green) and load capacitor voltage (blue).

In order to produce accurate simulation results, the value of HV energy-storage capacitor needs to be scaled down because the capacitor charging time (~min) is very long relative to the operational time period, *T* = 1/f (~ 19 µs in this case). Therefore, *C*_dc_ is set at 1 µF, to enable investigation of the circuit performance over the charging time, and verification of simulations with measurements. This is possible because, due to the current source nature of the circuit, its performance at a given percentage charge level is the same regardless of capacitance value. This is shown in the waveforms of Figs. 7 and 8 which show the simulation results of the DC/DC converter using full LTSpice models of all semiconductor switches for a 0.4 s run when *C*_dc_ = 1 µF and a 2 s run when *C*_dc_ = 5 µF (five times longer).

As mentioned, the PT initially sees the output load impedance as SC and tends towards OC as *C*_dc_ charges. The converter continues to charge *C*_dc_ until the maximum OC voltage is obtained for a given switching frequency and at given reflected load-impedance seen from PT output terminals. As can be observed, the circuit successfully charges the HV capacitor to 2 kV in approximately 0.12 s for *C*_dc_ = 1 µF and five times later at 0.6 s for 5 µF.

The output voltage waveform has a constant slope in both cases, confirming the circuit provides a constant charging current, while the PT resonant branch current is sinusoidal during charging (see expanded waveforms in Figs. 7 and 8b). In the full hardware prototype implementation, the input LV inverter bridge will be switched OFF as soon as 2 kV output is reached, thereby providing burst-mode control. As can be seen, the higher output voltage is possible, so long as the SiC voltage ratings are chosen to withstand such levels.

Calculations of the average PT power show that the rated power of 6 W is exceeded at an output voltage of ~ 1 kV at 53 kHz. The thermal performance of PTs was confirmed experimentally in previous works [15, 21] and it was found that PTs can be overloaded by up to 10 times their nominal power when operated under non-permanent excitation. Therefore, since the PT is not operating permanently at excess power levels and it can be controlled using burst-mode control, it is anticipated that the temperature rise within the PT will remain within its limits. Alternatively, to overcome this issue multiple PTs operating in parallel may be used to charge at a faster rate [17].

### Performance Comparison and Frequency Optimization

To illustrate the performance of the PT-based capacitor charger circuit at different operating frequencies and using WBG versus silicon power semiconductor devices, extensive LTSpice simulations were performed to compare the respective implementations as shown in Table 1. Simulations were run for 1 s to ensure *C*_dc_ was fully charged.

**Table 1:** WBG and Silicon-based implementation in LTSpice at *V*_dc_ = 24 V, *C*_dc_ = 1 µF

Device	WBG-based implementation	Silicon-based implementation
MOSFETs, T_1_ to T_4_	EPC2035, EPC Company, 60 V, 1.7 A	IRF540, Vishay/Siliconix, 100 V, 20A
Inductor, L_f_	22 μH	
Piezoelectric Transformer	ELM-610, 6 W	
HV diodes, D_1_ to D_4_	GAP3SLT33-220FP, GeneSiC Semiconductor, 3.3 kV, 300 mA	GP02D40, Vishay/Siliconix, 4 kV, 250 mA

The first observation was that for a given frequency, e.g. 53 kHz, the output voltage settles to 4.5 kV in the silicon-based implementation compared to 3.8 kV in the WBG-based implementation. This is most likely explained by differences in diode parasitic capacitances.

To compare the circuits’ performance for similar application conditions, results in Table 2 compare charging time and energy for a 1 µF load-capacitor charged to the same voltage; i.e. 3 kV with energy stored = 4.5 J, at an input voltage of 24 *V*_dc_ and operating frequencies of 52.5 kHz, 53 kHz, and 53.5 kHz. As can be seen, the WBG-based implementation has lower input energy and charging time thereby giving approximately 10% higher energy efficiency compared to the silicon implementation. Another important observation is that both implementations obtain highest efficiency at the switching frequency of 52.5 kHz indicating a higher voltage gain for the 1 µF load-capacitor as it is charged. Note that this frequency is the average resonant frequency between OC and SC conditions.

**Table 2:** Simulation results of input energy and charging time for WBG- and silicon-based implementation to charge 1 µF capacitor to 3 kV at input voltage of 24 VDC

Frequency (kHz)	WBG-based implementation		Silicon-based implementation	
	Input energy (J)	Charging time (ms)	Input energy (J)	Charging time (ms)
52.5	5	150	5.7	260
53	5.1	207	5.6	364
53.5	5.2	288	5.8	515

**Fig. 9:**
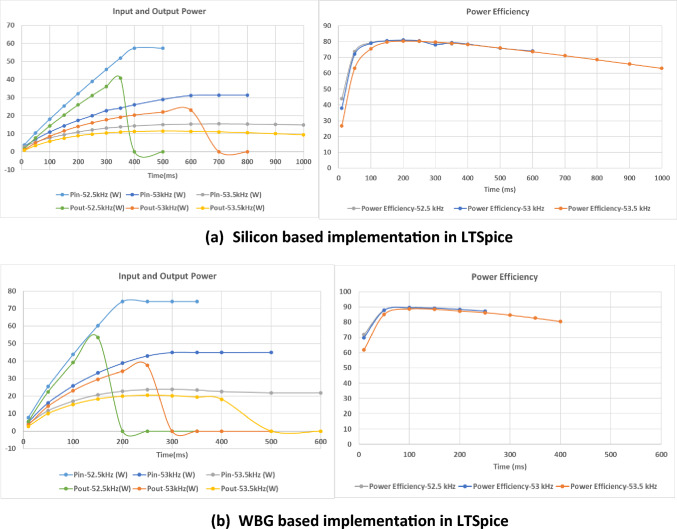
Average input power and corresponding output powers and efficiencies

Further analysis of the LTSpice simulation results was carried out in each case to get an understanding of the average circuit power and efficiency during the charging process. The average powers as well as efficiencies were calculated for four switching cycles at several output voltage levels and are shown as a function of charging time in Fig. 9. As already observed, operation at 52.5 kHz offers better charging efficiency for both implementations, but at the cost of increased input average power. Solutions for addressing high PT power levels have been discussed above; i.e. connecting several PTs in parallel and operating in burst mode. It is also seen that power efficiency is 10% more in the WBG implementation.

At all simulated operating frequencies, after the load-capacitor voltage settles at the OC voltage, all the input power is dissipated in the switching devices and the piezoelectric transformer, confirming that the PT must never be operated continuously when its output is OC.

### Stage II: HV Bi-Polar Pulse Generation

**Fig. 10:**
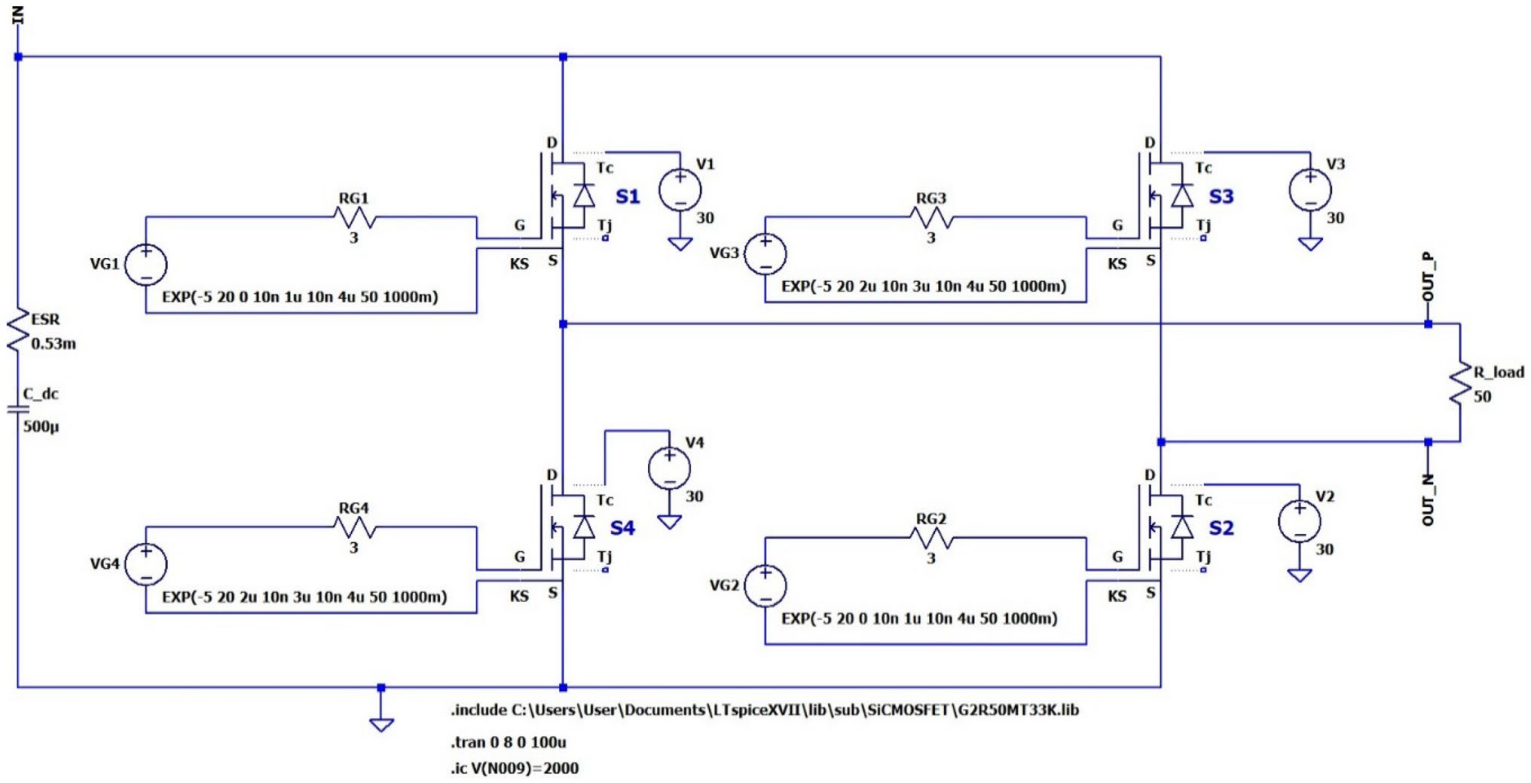
LTSpice schematic of full-bridge SiC HV inverter

Figure 10 shows a schematic diagram of the HV pulse generator using a charged HV capacitor as an input source in LTSpice simulation. While a low valued capacitor is used to illustrate the performance and characteristics of the capacitor charging in *Stage I* (based on available PTs and computational resources), the output pulse-generator stage is demonstrated using the 3.8 kV, 500 µF HV capacitor required for a standard electroporation treatment protocol. Based on the simulation results of *Stage I* with one single PT, the estimated time taken to charge a 500 µF capacitor to 2 kV is around 60 s. However, if five PTs are connected in parallel [17], the charge time would be approximately 12 s, which is significantly better than 2–3 min required for a recently proposed low-cost portable electroporator [34]. The SiC MOSFET G2R50MT33K rated at *V*_DS_ = 3.3 kV and *I*_DS_ = 44 A has been chosen for the output HV bridge-inverter (S_1_ to S_4_) which supplies HV bipolar pulses to the equivalent electrode load-resistance, chosen as *R*_load_ = 50 Ω.

**Fig. 11:**
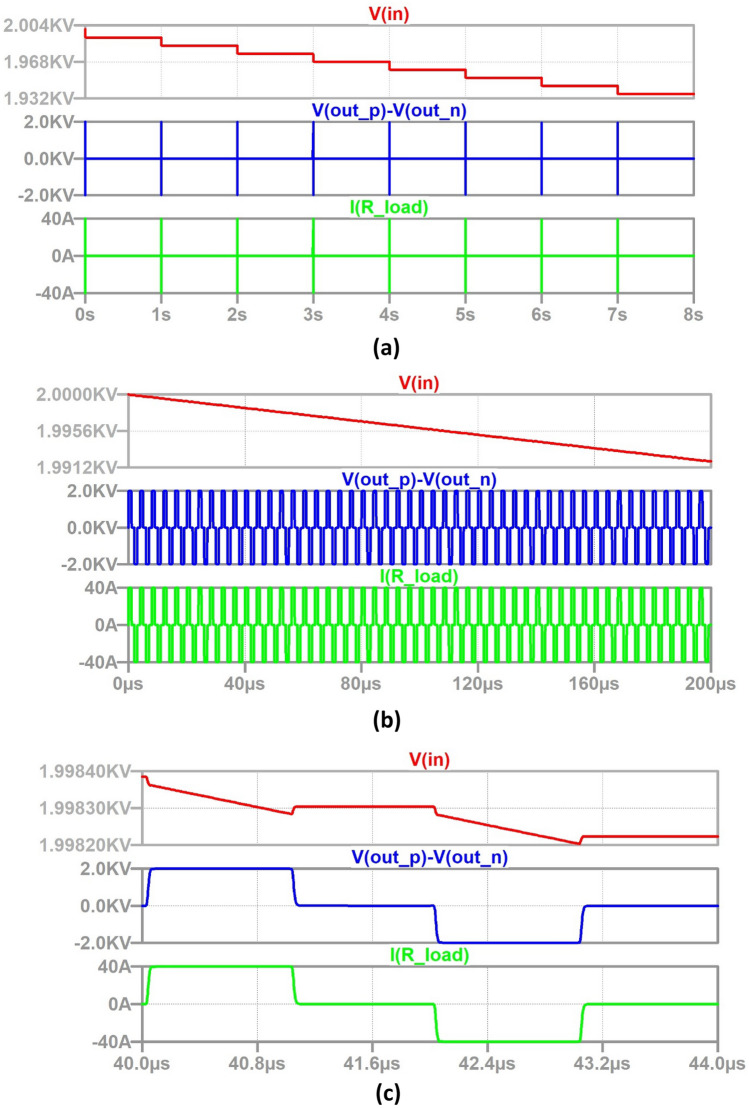
Output HV pulse waveforms: red trace is the terminal-voltage of the HV capacitor, blue trace is output voltage and green trace is load-current. **a** 8 bursts, **b** zoomed 1 burst, **c** zoomed 1 bipolar pulse

Figure 11a shows the overall simulation results of the HV pulse generator intended for a standard therapy from 0 to 8 s using full LTSpice models of all WBG switching devices and a pre-charged capacitor *C*_dc_ = 500 µF to 2 kV. It can be observed that the desired pulse pattern with ~ 2 kV amplitude is obtained, with the source capacitor voltage falling slightly during each discharge cycle. The voltage-drop at the end of 8 bursts is seen as approximately 70 V which matches the prediction of 3.5%. Figure 11b shows an expanded waveform of one burst of 50 bipolar pulses for the duration of 0 to 200 µs. Figure 11c shows an expanded waveform of one sample bipolar pulse for the duration of 40 to 44 µs.

In the case of a unipolar HV electroporation pulse waveform generator, gate-pulses would be supplied only to MOSFETs S_1_ and S_2_. A logic-circuit to provide gate trigger pulses to switches S_1_ to S_4_ can be designed to generate different HV pulse parameters.

## Experimental Results

The first converter stage, i.e. PT-based capacitor charger circuit has been built to verify it experimentally, using low-cost easily available commercial components as shown in Table 3. A discharge resistor of 100 MΩ is used. To maintain the output voltage within the range of available measurement equipment, the circuit has been tested at an input voltage of 2.5 V_dc_ at different frequencies to compare with trends predicted in simulations.

**Table 3:** Key hardware components

Component	Device
MOSFETs, ﻿T1 to T4	IRF540, Vishay/Siliconix 100 V, 20A, N-channel
Gate Drivers	IR2184PBF, Infineon
Series Inductor, *L*_f_	DR0810-393L, Coilcraft
PT	ELM-610, Eleceram
HV diodes, D1 to D4	HV5, 5 kV, 0.2A, DO-5 Diotec Semiconductor
Load capacitor, *C*_dc_	1uF, 3 kV, WIMA MKP1W041007K00KSSD

**Fig. 12:**
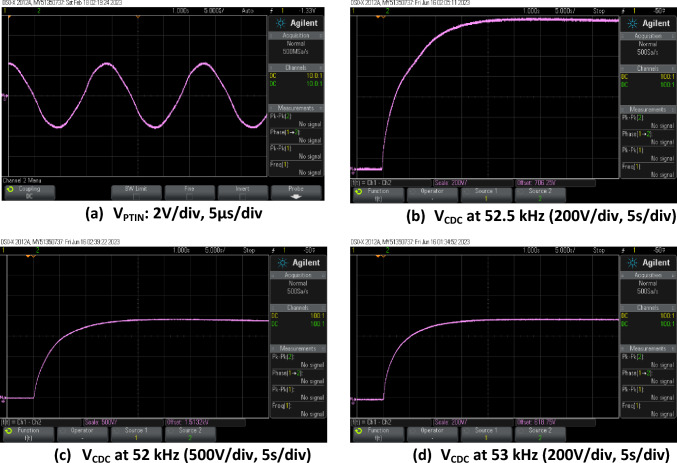
Experimental waveforms of **a** PT input voltage (*V*_PTIN_) at the end of the charging cycle and **b**–**d** voltage across load-capacitor (*V*_CDC_) during charging at given frequency

Figure 12 shows the measured input to the PT at 53 kHz and output voltage waveforms measured directly across the load capacitor *C*_dc_ using two HV probes (HV250: Oscilloscope Probe, Passive, 300 MHz, 2.5 kV, 100:1) for three different frequencies. Summary results are presented in Table 4. It can be observed from the measured output voltage waveforms, that the DC link capacitor *C*_dc_ is successfully charged to different voltages depending upon the operating frequency and overall voltage gain of the circuit. As predicted in simulations, there is an optimum frequency at which the fastest charging performance and highest voltage gain is found; i.e. 52 kHz in this case which is between the SC and OC resonant frequencies discussed earlier.

**Table 4:** Summary of the experimental results for the input voltage of 2.5 *V*_dc_ at different operating frequency

Frequency (kHz)	Output voltage (V)	Charging time (s)	Charging time to 300 V (s)
52	1900	20	0.70
52.5	1460	25	0.75
53	760	25	1.4

**Fig. 13:**
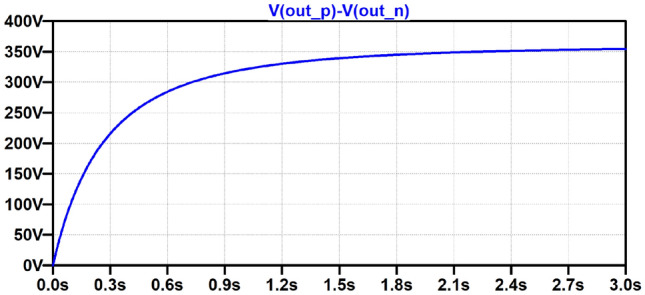
Simulated output voltage during charging at 53 kHz

Figure 13 shows simulation results of the same circuit at 2.5 *V*_dc_ at 53 kHz for 3 s, which is the longest time possible with available computing resources. It is found that the capacitor is charged to 350 V at this time, which compares with 450 V in measurements. To compare with the trends predicted in the simulation results of Table 2, measured results of charging time to 300 V are included in Table 4. An overall increase in charging time with frequency is observed that approximately doubles from 52 to 53 kHz, similar to trends found in simulations from 52.5 to 53.5 kHz.

While there is a mismatch between simulation and measurement results, the overall charging characteristic is confirmed, and the trends of increasing charging time with operating frequency around the PT resonant frequency match. The mismatch is likely due to the simulation model of the PT [3] which has a tolerance of ± 3% on its resonant frequency. In addition, it has previously been found that the equivalent circuit model offers higher accuracy for low impedance loads [16], while a wide range of impedances up to OC are considered here.

## Discussion

This paper proposes the application of a PT-based power converter architecture to generate HV pulses for electroporation. PT-based power converters are very popular for high-gain, HV power conversion applications such as CCFL inverters for LCD backlight, laser-beam printers, ozone generation etc. Therefore, the reduction in size, weight and form-factor achieved in those applications are expected to translate to the proposed application in medical electroporation, where the additional advantage of potential elimination of magnetic components may be particularly important.

Wide-bandgap (WBG) power semiconductor devices have been applied for the PT-based HV capacitor charging and subsequent HV bipolar pulse generator at the output. LTSpice simulation results successfully demonstrate the effectiveness of the proposed converter architecture to generate 2 kV, 40 A bipolar pulses with only 24 *V*_dc_ input. The ratio of output pulse-voltage amplitude to input DC voltage is around 84, which is comparable to recently demonstrated power converter architectures for medical electroporation in the literature.

The experimental results of a PT-based capacitor charger demonstrate the proof-of-concept of circuit operation of Stage I. A complete hardware implementation of the whole power converter that includes combined operation of Stage I and Stage II with a closed loop control is under development.

The proposed architecture employs a reduced number of semiconductor switches thereby offering improved reliability and reduced cost. It also offers the potential to provide a magnetic-free solution, which may be important in biomedical environments where a HV pulse generator during electroporation may need to operate under the influence of external magnetic fields such as in real-time magnetic resonance electrical impedance tomography (MREIT) [18, 19] for measuring electrical conductivity during electroporation.

The PT employed is not custom designed or optimized for this new application and if done so, better performance metrics are expected.
